# Pathogenicity of *Purpureocillium lilacinum* and *Clonostachys rosea* against fall armyworm (*Spodoptera frugiperda*) under laboratory conditions

**DOI:** 10.1371/journal.pone.0334730

**Published:** 2026-03-16

**Authors:** Abel Jonathan Mussa, Sija Kabota, Joseph O Ruboha, Martin John Martin, Maulid W Mwatawala

**Affiliations:** 1 Department of Crop Science and Horticulture, Sokoine University of agriculture, Morogoro, Tanzania; 2 Research, Consultancy and Publication Unit, National Sugar Institute (NSI), Kidatu-Morogoro, Tanzania; 3 Department of Agricultural Sciences, Sokoine University of agriculture, Mpanda-Katavi, Tanzania; 4 Institute of Pest Management IPM, Sokoine University of agriculture, Morogoro, Tanzania; UNSTIM: Universite Nationale des Sciences Technologies Ingenierie et Mathematiques, BENIN

## Abstract

**Background:**

Fall armyworm, *Spodoptera frugiperda* (J.E. Smith) threatens staple crops across Africa. Integrating entomopathogenic fungi into Integrated Pest Management (IPM) offers a sustainable alternative to sole reliance on insecticides. This study quantified the pathogenicity of *Purpureocillium lilacinum* and *Clonostachys rosea* against *S. frugiperda* under controlled conditions.

**Methods:**

Second-fifth instar larvae and eggs were exposed to 1 × 10^7^, 1 × 10^8^, and 1 × 10^9^ conidia mL^-1^ of each fungus; sterile water served as control. Mortality was recorded over 3–9 days after treatment (DAT); feeding reduction was measured gravimetrically. Larval mortality was analyzed with GLMs/GLMMs (binomial-probit); feeding reduction by ANOVA/Tukey; LD50 and LT50 were estimated from dose-response models.

**Results:**

Larval mortality was significantly affected by concentration × time interaction and declined with advancing larval stage. Peak larval mortality was reached at a concentration of 1 × 10^9^ conidia mL^-1^ at 9 DAT. Feeding consumption reduction was significantly affected by larval instar, EPF species, and instar × concentration. Feeding reduction reached 60−74% in early instars at the highest dose. Egg mortality was primarily concentration-dependent with maximum values (up to 82% and 88% for *P. lilacinum* and *C. rosea*, respectively) at the dose of 1 × 10^9^ conidia mL^-1^ highest dose. Our findings supported the study hypothesis that efficacy of entomopathogenic fungi against *S. frugiperda* is primarily driven by interaction of spore concentration and exposure time across the host developmental stages, rather than the interaction of fungal species. The consistent susceptibility of early instars and the strong concentration-dependent responses highlight the functional potential of these native fungi as biologically relevant components of sustainable IPM strategies.

**Conclusions:**

Native *P. lilacinum* and *C. rosea* display dose‑, stage‑, and time‑dependent pathogenicity and feeding suppression against *S. frugiperda*. These species are promising candidates for IPM; field validation and formulation optimization are the next steps.

## Introduction

Fall armyworm (*Spodoptera frugiperda* J.E. Smith) is one of the most destructive invasive pests threatening food security in Sub-Saharan Africa. Native to the tropical and subtropical regions of the Americas, *S. frugiperda* was first detected in Africa in 2016 [1] and subsequently reported in Tanzania in 2017 [2,3]. Since its invasion, the pest has spread rapidly across multiple agroecological zones, infesting major crops such as maize, sorghum, rice, and legumes. Yield losses attributed to *S. frugiperda* are severe and vary by country and production system. In Kenya, for example, maize yield losses range from 32–47%, while in Ethiopia they average around 32% [4]. These reductions are equivalent to thousands of tons of maize annually, and because maize is the primary staple crop in much of Sub-Saharan Africa, the implications for food security are profound. At the regional scale, economic losses have been estimated to exceed US$13 billion annually [2,5]. The destructive capacity of *S. frugiperda* stems from its larval feeding habit, in which caterpillars aggressively consume foliage, whorls, and reproductive structures, leading to defoliation, stalk breakage, and poor grain filling. In heavily infested fields, infestation levels can reach 100%, resulting in near-total crop failure [6]

In Tanzania, management of *S. frugiperda* has primarily relied on the application of synthetic insecticides, valued for their rapid action and availability [3,5,7]. Nonetheless, continuous reliance on chemical control has raised critical concerns. Resistance to active ingredients has already been documented in several countries, reflecting the pest’s genetic adaptability and high reproductive potential. Moreover, synthetic insecticides pose risks to beneficial arthropods, pollinators, and natural enemies, thereby disrupting ecological balance and resilience of farming systems [8–10]. Environmental pollution, human health hazards, and escalating input costs further constrain the sustainability of insecticide-based strategies. These challenges underscore the urgent need for ecologically sound and locally adaptable alternatives to chemical control, particularly the development of biological control agents that are compatible with integrated pest management (IPM).

Entomopathogenic fungi (EPFs) have emerged as an important component of biological pest management. These fungi are naturally occurring insect pathogens with the ability to infect and kill more than 700 insect species across different orders [11]. Unlike other microbial control agents that often require ingestion, EPFs are unique in their mode of action: they penetrate directly through the insect cuticle via enzymatic activity, colonize the hemocoel, and proliferate within the host, ultimately causing death through nutrient depletion and toxin production [12–15]. This capacity enables them to infect foliar as well as soil-dwelling pests, providing a wide ecological reach. Several genera including *Beauveria*, *Metarhizium*, *Isaria* (syn. *Cordyceps*), and *Purpureocillium* have been extensively studied, leading to the development of commercial mycoinsecticides [16,17]. Their ecological safety, compatibility with other IPM components, and potential to reduce pesticide dependence make them central to sustainable and agroecological pest management strategies.

Recent advances in entomopathogenic fungi research have reinforced their promise as biological control tools. For instance, Shehzad et al. [18] emphasized the ecological safety of EPFs as natural biocontrol agents, while Vivekanandhan et al. [19], and Gielen et al. [20] highlighted EPFs as effective and sustainable options, documenting continued innovation in formulation, application methods, and strain improvement. Both laboratory and field studies have repeatedly confirmed their insecticidal efficacy against major agricultural pests. *Beauveria* and *Metarhizium* species, in particular, have demonstrated consistent performance against lepidopteran and coleopteran pests, including *S. frugiperda*, across diverse regions [21–28]. Alongside these well-established fungi, new candidates are gaining attention. *Purpureocillium lilacinum* (Thom) and *Clonostachys rosea* (Link) are emerging as multifunctional fungi with dual potential in agriculture. Traditionally recognized for their antagonism against soilborne plant pathogens, they are increasingly acknowledged for their capacity to infect and suppress insect pests [29–33]. Laboratory trials in Pakistan with *P. lilacinum* demonstrated significant insecticidal activity [34–37], while multiple studies have reported insecticidal efficacy of *C. rosea* against a wide range of pests [31,38–40].

Both *P. lilacinum* and *C. rosea* are cosmopolitan species, naturally occurring in soils, decaying organic matter, nematodes, and insect cadavers [32,41,42]. Beyond their insecticidal action, they contribute to plant growth promotion and biofertilization by enhancing nutrient uptake and suppressing plant pathogens [29,43,44]. These multifunctional attributes make them attractive candidates for integration into pest and soil health management programs. However, despite their global distribution, research on these fungi in Tanzania remains underdeveloped. While both species have been reported in the country, most studies focus on imported fungal formulations rather than exploring the diversity and efficacy of native isolates. Moreover, some locally available fungi have been found pathogenic to crops and remain unvalidated for their potential mycotoxin production, raising safety concerns [9,10,45]. The systematic isolation, characterization, and pathogenicity testing of native fungal strains against *S. frugiperda* and other insect pests have not yet been undertaken at scale, leaving a major gap in the development of homegrown biocontrol solutions.

No published study in Tanzania has yet evaluated the pathogenicity or virulence of native isolates of *P. lilacinum* and *C. rosea* against *S. frugiperda*. Given the pest’s economic importance and the limitations of current insecticide-based control strategies, exploring the pathogenic potential of these fungi is both timely and necessary. Establishing the efficacy of native isolates could expand the repertoire of EPFs available for local pest management, reduce dependence on imported products, and support the development of context-specific biological control strategies.

This study evaluated the pathogenicity and virulence of native *P. lilacinum* and *C. rosea* isolates against multiple stages of *S. frugiperda* under laboratory conditions, focusing on larval and egg mortality as well as feeding reduction at different spore concentrations and exposure times. The findings broaden the diversity of EPFs available for pest control in Tanzania, clarify stage-specific susceptibility critical for management design, and provide baseline data for optimizing EPF-based interventions within IPM frameworks. By diversifying control options, the study supports reduced reliance on insecticides, improved resistance management, and more sustainable agriculture.

It was hypothesized that native isolates of *P. lilacinum* and *C. rosea* would exhibit dose, stage, and time-dependent pathogenicity against *S. frugiperda*. Specifically, younger larval instars were expected to show higher susceptibility due to weaker cuticular defenses and less developed detoxification mechanisms, while older larvae were predicted to be more resilient. Furthermore, it was anticipated that higher spore concentrations and longer post-treatment periods would result in greater mortality and stronger suppression of feeding activity.

## Materials and methods

### Study area

The study was conducted from August 2024 to February 2025 in the Mycological and Molecular Laboratories, Department of Crop Science and Horticulture, Sokoine University of Agriculture (SUA), Morogoro, Tanzania (6.8520°S, 37.6576°E). Morogoro Municipality is located on the lower slopes of the Uluguru mountains at an elevation of approximately 500–600 m above sea level. The area experiences a tropical sub-humid climate with mean annual rainfall ranging from 800 to 1,200 mm, distributed in two rainy seasons: the short rains from October to December and the long rains from March to May. Average annual temperatures range between 18°C and 30°C, with relatively cooler conditions during the rainy season and warmer conditions during the dry season [46]. The area is characterized by steep slopes, deeply dissected valleys, and diverse vegetation ranging from lowland woodlands to montane and cloud forests with diverse agricultural systems [47].

### Collection and rearing of test insect

*Spodoptera frugiperda* larvae were collected from maize fields using culture vials lined with cotton wool and reared following protocol of Idrees et al. [22] with minor modification. Preferably, fourth to sixth instar larvae were selected and transported to the laboratory for rearing. In the laboratory, larvae were transferred into sterile, aerated plastic containers and provided with fresh, sterile maize leaves. The leaves were replaced every 24 hours to maintain hygiene and ensure adequate nutrition. Once pupation occurred, the containers were supplemented with sterilized sand to facilitate pupal development. Pupae were then transferred to aerated rearing cages to allow for adult emergence.

Emerging adult moths were maintained in cages and supplied with a 10% honey solution soaked in cotton wool for sustenance. Potted maize plants were introduced into the cages to serve as oviposition substrates. Upon hatching, larvae were collected and transferred to new sterile plastic containers, provided with fresh maize leaves, and maintained under controlled laboratory conditions: 25 ± 2°C temperature, 65 ± 5% relative humidity, and a 12:12 h light: dark photoperiod. The colony was maintained for at least two successive generations to obtain sufficient numbers of target-stage larvae for bioassays [22]. Only second to fifth instar larvae were used for bioassay tests because first instars are extremely fragile and prone to mortality due to mishandling. On the other hand, sixth instars are less susceptible to EPF due to a thicker cuticle and could pupate during the assay, potentially confounding mortality assessments; thus, the selected instars provided an optimal balance between susceptibility and experimental reliability [48].

### Preparation of fungal suspension for bioassay

Entomopathogenic fungal species were obtained from soil samples collected along the slopes of the Uluguru Mountains through selective media isolation following the established protocol by Humber [49] and screened for pathogenicity. The pathogenic isolates were confirmed using morphological characters and ITS rDNA sequencing, which were compared against GenBank references. Single-spore of pathogenic isolates were cultured on Potato Dextrose Agar (PDA) and incubated at 25°C in darkness for two weeks. Conidia were harvested in sterile double-distilled water, filtered through sterile gauze to remove mycelial debris, and gently mixed to ensure a uniform suspension. Spore concentration was determined using a hemocytometer, and viability was confirmed before use. A stock suspension of 1 × 10^8^ conidia mL^-1^ was prepared and diluted or concentrated to 1 × 10^7^, 1 × 10^8^, and 1 × 10^9^ conidia mL^-1^ for bioassays [48].

### Bioassay of fungal species against immature stages of *S. frugiperda*

A pathogenicity test bioassay was conducted using a Completely Randomized Design (CRD) with three replicates per treatment to evaluate the efficacy of EPF species against immature stages of *S. frugiperda*. Each group of 10 larvae (second to fifth instar) and 20 eggs attached to maize leaves were separately placed in sterile, aerated plastic container as described by [48] and [50]. Each group was uniformly sprayed once with 10 mL of each fungal suspension at concentrations of 1.0 × 10^7^, 1.0 × 10^8^, and 1.0 × 10^9^ conidia mL^-1^ using handheld sprayer, while a negative control received sterile distilled water. Sterile paper towels were placed beneath maize leaves in each container to absorb excess spray solution and removed thereof after 24 hours, and fresh, sterile maize leaves were provided daily under replacement as a food source to larvae. All test units were maintained at 25 ± 2°C, 65 ± 5% relative humidity, and a 12:12 h light: dark photoperiod throughout the observation period. Larval mortality was recorded every 24 hours for nine days post-inoculation, and egg mortality was determined by counting hatched and unhatched eggs nine days after treatment. Dead insects were removed, isolated in sterile conditions, and examined for typical signs of mycosis induced by EPFs.

### Assessment of feeding consumption reduction of *S. frugiperda* larvae

Feeding activity of *S. frugiperda* larvae was evaluated following the methodology of Idrees [22], with minor modifications to suit experimental conditions. Each group of 10 larvae from each of the 2^nd^, 3^rd^, 4^th^, and 5^th^ instars were separately provided with 7 grams of fresh, sterile, and untreated maize leaves daily. To maintain consistency and prevent leaf desiccation or contamination, the maize leaves were replaced every 24 hours.

Feeding activity was quantified by measuring the weight of maize leaves before and immediately after the 24-hour feeding period. The difference in weight represented the amount of leaf material consumed by the larvae, allowing for an indirect assessment of the physiological effects of EPF treatments on larval feeding behavior. This approach effectively monitored larval consumption rates and served as a crucial parameter for evaluating the biocontrol potential of the fungal species.

To determine the impact of EPF treatments on feeding, the percentage reduction in feeding consumption was calculated using the formula:


$$\text{Feeding Consumption Reduction }(\%)=[({\text{W}}_{\text{c}}-{\text{W}}_{\text{t}})/{\text{W}}_{\text{c}}]\times \text{100}$$

where:

W_c_ = weight of leaf material consumed by the control (untreated) larvae

W_t_ = weight of leaf material consumed by the treated larvae

### Ethics statement

This study protocol was reviewed and approved by the Ethics Review Board of the College of Agriculture, Sokoine University of Agriculture (SUA), Tanzania (Approval Number: SUA/DPRTC/MCS/D/2022/0021/07). Written informed consent to access and conduct research at the field sites and laboratory was waived by the Ethics Review Board of Sokoine University of Agriculture, which owns and manages all land and facilities where the study was carried out. As the study was conducted entirely within university-owned properties, no additional permits from external authorities were required. This study did not involve human participants, animals or human participants’ data; therefore, written informed consent was not required.

### Statistical data analysis

All data on mortality and feeding reduction were analyzed using R version 4.4.3 [51]. Normality of feeding reduction data was assessed using the Shapiro-Wilk test. When assumptions were met, analysis of variance (ANOVA) followed by Tukey’s HSD post hoc test (α = 0.05) was used.

Larval mortality data, expressed as proportions, were analyzed using generalized linear models (GLMs) with a binomial error distribution and probit link function. To account for repeated measures and fungal species variation, generalized linear mixed-effects models (GLMMs) were fitted with conidial concentration and time after treatment as fixed effects and replicates nested within species as a random effect.

Lethal dose (LD_50_) and lethal time estimates were obtained using the dose.p function from the MASS package. Model significance was evaluated with Wald z-tests and Type III χ² tests using the car::Anova function. Model fit and residual diagnostics were assessed using the DHARMa package.

## Results

### Effect of *P. lilacinum* and *C. rosea* on larval mortality of *S. frugiperda*

**Fig 1:**
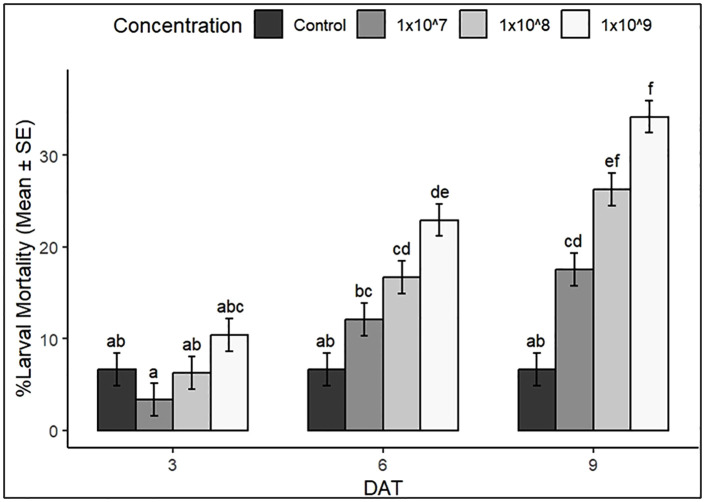
Effect of conidial concentration and Days After Treatment (DAT) on mortality of *S. frugiperda* larvae. Different letters above bars indicate significant differences among concentrations at each DAT (Tukey’s HSD, p < 0.05). Mortality also increased significantly with DAT (p < 0.001), reflecting the time-dependent virulence of the entomopathogenic fungi. Error bars represent standard error of the mean (SEM).

We observed a significant effect of concentration × time interaction on larval mortality (*p* = 0.01; Table 1). This indicates that the effect of spore concentration on larval mortality was significantly dependent on the duration of exposure. Increasing spore concentration from 0 to 1 × 10^9^ conidia mL^-1^ caused a significant rise in mortality, with mortality reaching peak at 9 DAT. Post hoc analysis (Tukey HSD) showed that mortality at 9 DAT was significantly higher than that recorded at 3 and 6 DAT (Fig 1). Overall, mortality was significantly higher at 9 DAT across all concentrations and lowest at 3 DAT (*p* < 0.001).

**Table 1:** Analysis of variance (ANOVA) results for the effects of spore concentration, larval instar stage, and time after treatment, along with their interactions on *S. frugiperda* larval mortality caused by EPFs.

Source of variation	χ²	Df	p-value
Concentration (C)	70.1107	13	***
Instar Stage (I)	52.11186	13	***
Days After Treatment (DAT)	66.65455	8	***
Species (S)	7.836619	5	ns
C: S	1.991494	7	ns
**C: DAT**	15.90452	10	*
C: I	4.006428	16	ns
S: DAT	1.53809	4	ns
S: I	1.915249	7	ns
DAT: I	2.711469	11	ns
C: S: DAT	0.763631	7	ns
C: DAT: I	2.132113	19	ns
S: DAT: I	1.931265	7	ns
C: S: I	1.166897	10	ns
C: S: DAT: I	1.248728	18	ns

Notes: Bold text indicates significant interaction effects. Asterisks indicate the level of significant difference: *** p < 0.001, ** p < 0.01, * p < 0.05, ns p ≥ 0.05.

**Fig 2:**
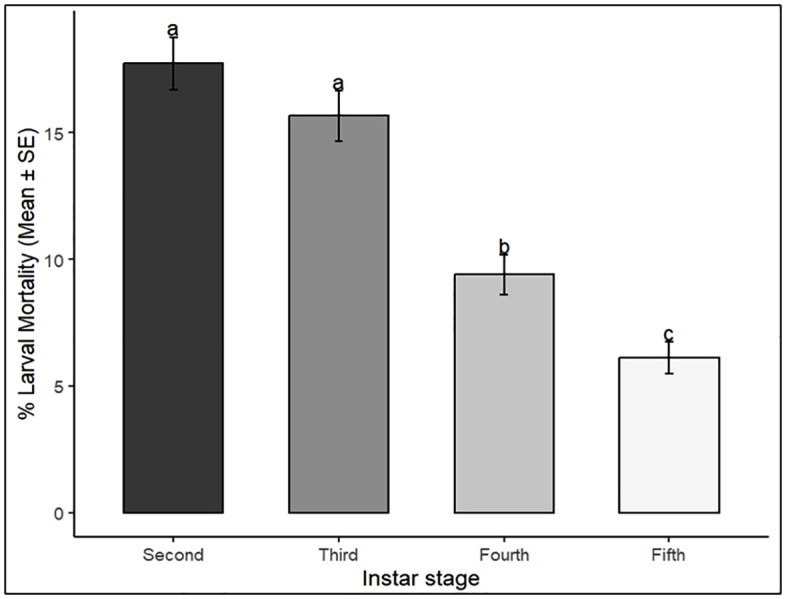
Larval mortality of *S. frugiperda* across instar stages following treatment with *C. rosea* and *P. lilacinum.* Bars show mean ± SE. Different letters above bars indicate significant differences among instars (Tukey’s HSD, *p* < 0.05).

Mortality also significantly varied with larval instar stage (*p* < 0.001), with second and third instars being more susceptible than fourth and fifth instars, irrespective of concentration and time (Fig 2). The main effect of fungal species was not significant (*p* > 0.05), although *C. rosea* caused slightly higher mortality (48.5% at 9 DAT) compared to *P. lilacinum* (43.7%).

**Fig 3:**
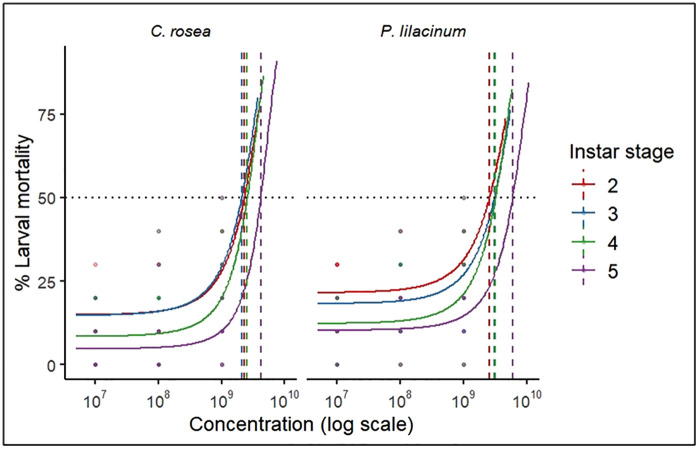
Dose-response curves of *S. frugiperda* larval mortality across instar stages following treatment with EPFs. Curves fitted with probit regression.

Probit analysis showed that each additional day post-treatment increased mortality by 3−4%, while a tenfold increase in dose raised mortality by approximately 7% (S1Table). Conidial concentration and exposure time significantly affected mortality, with minimal random variation. The lethal dose (LD_50_) was estimated at 1.6 × 10^12^ conidia mL^-1^, which representing the average estimated virulence across all tested instars. The median lethal time (P_50_) decreased from 15.4 to 11.8 days with increasing dose, indicating clear dose- and time-dependent virulence. Dose-response curves further showed that second and third instars required lower conidial concentrations to reach LD_50_ than fourth and fifth instars (Fig 3).

### Effects of *P. lilacinum* and *C. rosea* on feeding consumption reduction of *S. frugiperda* larvae

**Fig 4:**
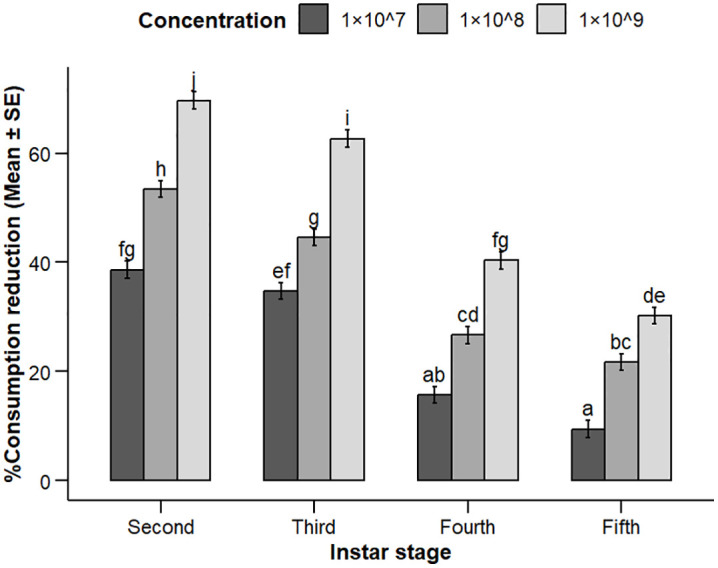
Feeding reduction (%) of *S. frugiperda* larvae treated with EPFs at three concentrations (1 × 10^7^, 1 × 10^8^, and 1 × 10^9^ conidia mL^-1^). Bars show mean ± SE. Different letters indicate significant differences (*p* < 0.05, Tukey’s HSD).

Feeding consumption reduction was strongly influenced by the interaction between concentration and larval instar stage (concentration x instar stage, *p* < 0.001), indicating that the effect of concentration on feeding depends on the developmental stage of the larvae. In contrast, fungal species-related interaction effects were not significant (*p* > 0.3). The main effects of spore concentration, fungal species and instar stage were also significant (all *p* < 0.001; Table 2). A clear concentration-instar dependent effect was observed (Fig 4), at 1 × 10^9^ conidia mL^-1^, *C. rosea* caused the highest reduction (74% in second instars, 66% in third instars). *P. lilacinum* achieved maximum reductions of 66% (second instars) and 60% (third instars) at the same dose. Both fungi were less effective against fourth and fifth instars (<45% and <35%, respectively).

**Table 2:** ANOVA summary table of the effects of different spore concentration of EPF species on feeding consumption reduction in different instars of *S. frugiperda.*

Source	Df	F-value	p-value
Concentration (C)	2	999.94	***
Instar stage (I)	3	1661.39	***
Species (S)	1	28.03	***
C × I	6	30.45	**
C × S	2	0.16	ns
I × S	3	0.04	ns
C × I × S	6	6.55	ns

Notes: ANOVA performed on arcsine-transformed proportions. Asterisks indicate the level of significant difference: *** p < 0.001, ** p < 0.01, * p < 0.05, ns p ≥ 0.05.

### Effects of *P. lilacinum* and *C. rosea* on egg mortality

ANOVA showed that spore concentration had a highly significant effect on egg mortality (*p* < 0.001), with mortality increasing at higher concentrations of both fungal species (Table 3). Neither the main effect of fungal species (*p* = 0.2291) nor its interaction with concentration was significant (*p* = 0.9707), indicating that egg mortality was primarily determined by spore concentration, rather than fungal species.

**Table 3:** ANOVA summary table of the effects of different spore concentration of EPF species on eggs mortality of *S. frugiperda.*

Source of Variation	F-value	Df	p-value
Concentration	186.47894	3	***
Species	1.4463557	1	ns
Concentration: Species	0.2409136	3	ns

Notes: Asterisks indicate the level of significance: *** p < 0.001, ns = p ≥ 0.05.

**Fig 5:**
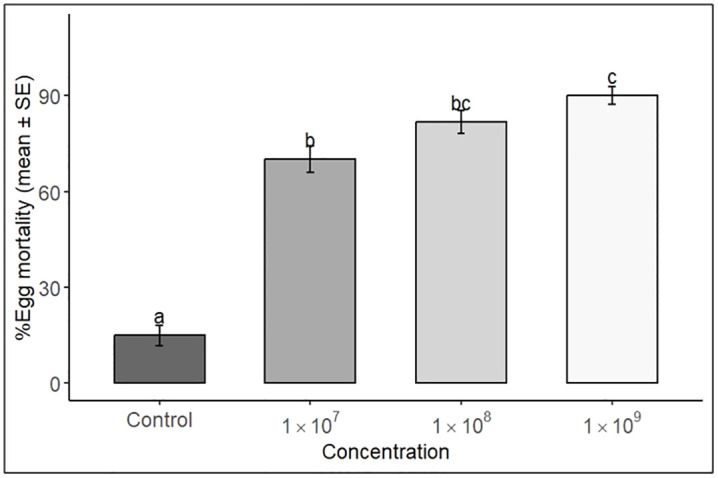
Egg mortality of *S. frugiperda* in response to *C. rosea* and *P. lilacinum* treatments at different concentrations. Bars show mean ± SE. Different letters indicate significant differences among concentrations (*p* < 0.05, Tukey’s HSD).

At 1 × 10^9^ conidia mL^-1^, *C. rosea* caused maximum egg mortality of 88.3%, while *P. lilacinum* achieved 81.7%. Mean egg mortality at 1 × 10^9^ conidia mL^-1^ was significantly higher than at lower concentrations and the control, as indicated by Tukey HSD grouping letters (Fig 5).

## Discussion

Our results confirm that both *Purpureocillium lilacinum* and *Clonostachys rosea* possess measurable insecticidal potential against *S. frugiperda*, with performance varying by developmental stage, fungal species, and conidial concentration. As expected, larval mortality increased with higher inoculum levels, while early instars were more susceptible than older larvae, demonstrating a clear dose- and age-dependent virulence pattern. Similar findings have been reported for other entomopathogenic fungi. In Tanzania, *Metarhizium anisopliae*, *Fusarium* spp., and *Beauveria bassiana* caused greater mortality in early instars of *S. frugiperda*, with effects enhanced by higher doses and longer exposure times [9,10,52]. Comparable stage- and dose-dependent responses have also been documented in Kenya and Ethiopia, where *Metarhizium* spp. and *Beauveria* spp. produced significant larval mortality [23,26].

In our assays, *P. lilacinum* showed particularly high pathogenicity against eggs and neonates, with mortality exceeding 80% at 1 × 10^9^ conidia mL^-1^, but efficacy declined against later instars, often falling below 50% at the same concentration. This stage-specific dose-response pattern has been widely observed. Laboratory bioassays confirmed that *S. frugiperda* eggs and neonates are highly susceptible, with mortality above 95% at 10^6^−10^8^ conidia mL^-1^, while 1^st^-2^nd^ instars required higher concentrations, yielding LD₅₀ values of 1.38 × 10^8^–2.56 × 10^8^ conidia mL^-1^ by day 7 [30,37]. In Egypt, Suzan et al. [53], similarly reported larval mortality of 37.5% to 68.5% across a gradient of 10^5^−10^9^ conidia mL^-1^. Beyond *S. frugiperda*, *P. lilacinum* has demonstrated strong activity against other lepidopterans: *Galleria mellonella* larvae exhibited LD_50_ values as low as 3.1 × 10^4^ conidia mL^-1^, with LT_50_ around 1−2 days at 10^8^ conidia mL^-1^ [54]. while high mortality has also been reported in *Tuta absoluta* and *Spodoptera litura* [35]. The fungus is also effective against insect orders such as Hemiptera and Diptera, although feeding larvae in these groups typically require higher doses [33,36,55–57]. Importantly, *P. lilacinum* produces sublethal effects even at lower inoculum levels, including delayed larval development, reduced pupal weight, and impaired adult emergence [58]. These additional impacts suggest that its influence extends beyond direct mortality.

The value of *P. lilacinum* is further enhanced by its activity against plant-parasitic nematodes, particularly *Meloidogyne* spp., where suppression is dose-dependent [59,60]. It is also capable of colonizing plants as an endophyte, thereby providing systemic protection against both insects and pathogens, while stimulating plant growth [58,59]. Its production of secondary metabolites, such as leucinostatins and paecilotoxins, likely underpins both insecticidal activity and feeding inhibition [55,61]. Together, these attributes position *P. lilacinum* as a versatile biocontrol agent that is most effective when applied early in pest infestations, with strong potential for integration into diversified pest management programs.

Although direct studies on *C. rosea* against *S. frugiperda* are lacking, evidence from other pests highlights its significant insecticidal capacity. In lepidopterans such as *G. mellonella* and *T. absoluta*, *C. rosea* caused up to 97% mortality [31,62]. In stored-product beetles including *Callosobruchus maculatus*, *Trogoderma granarium*, and *Tribolium castaneum*, mortality rates of 71−76% were achieved at 10^8^ conidia mL^-1^, with LT_50_ values of 4.8–5.0 days [63]. Against hemipterans, moderate to high activity has been reported: *Diaphorina citri* showed up to 47% mortality at 10^8^ conidia mL ⁻ ¹, while *Amritodus atkinsoni* reached nearly 97% mortality at 3 × 10^8^ conidia mL^-1^ after 7 days [39,40]. Efficacy has also been demonstrated across Coleoptera, Hemiptera, and Hymenoptera in other regions [38,64–68]. In Tanzania, *C. rosea* has been naturally isolated from *S. frugiperda* cadavers [41], confirming ecological association, though its direct virulence against this pest remains untested. Beyond insect pathogenicity, *C. rosea* plays a dual role as a mycoparasite, antagonizing plant pathogens such as *Botrytis cinerea* and *Fusarium* spp. in a dose-dependent manner [29,34,69], while also promoting plant growth through endophytic colonization [31,44]. Its mechanisms of action include direct infection of insect hosts, rhizosphere competition, and the production of antifungal and insecticidal metabolites [61], enhance its versatility in agroecological systems. Furthermore, its compatibility with IPM programs and its safety to beneficial insects support its suitability for sustainable agriculture [70]. However, compared with *P. lilacinum* and classical genera such as *Beauveria* and *Metarhizium*, insect-focused dose-response studies of *C. rosea* remain scarce, highlighting a key knowledge gap and an opportunity for future research.

Both fungi displayed strong stage-dependent efficacy, with eggs and neonates consistently more vulnerable than feeding larvae. LD_50_ values for early developmental stages were often an order of magnitude lower than for later instars [30,53]. This pattern reflects fundamental differences in insect physiology: younger larvae possess softer cuticles, lower levels of detoxification enzymes, and weaker immune responses, whereas older instars develop thicker cuticles and more robust defenses that limit fungal penetration and proliferation [19,71,72]. Such age-dependent susceptibility has been consistently observed across other entomopathogenic fungi [50,73], reinforcing the importance of applying these fungi early in pest infestations to maximize biocontrol efficacy.

The performance of *P. lilacinum* and *C. rosea* should also be viewed in the broader context of entomopathogen research. Although *Beauveria* and *Metarhizium* remain the most widely studied and commercialized fungi [18], emerging evidence indicates that less-studied taxa can complement or, under certain circumstances, outperform them. Both *P. lilacinum* and *C. rosea* are ecologically relevant, occurring in diverse soils and frequently associated with insect cadavers. Their ability to induce multiple outcomes including direct mortality, feeding inhibition, reduced fecundity, and developmental delays further enhances their potential. Nonetheless, gaps remain in understanding their persistence, dispersal, and sensitivity to environmental factors such as UV radiation and humidity, which often constrain field performance [20].

In Africa, integrated biological control approaches are gaining attention. Ngangambe et al. [9], demonstrated enhanced suppression of *S. frugiperda* when entomopathogenic fungi were combined with parasitoids, compared with single-agent applications, suggesting valuable synergistic effects. Such findings highlight the potential for *P. lilacinum* and *C. rosea* to be deployed not only as stand-alone agents but also as components of multi-enemy strategies tailored to smallholder farming systems. Moreover, ecological approaches such as push-pull technology in Kenya have been highly effective against *S. frugiperda*. By intercropping maize with *Desmodium* (push) and planting Napier or Brachiaria grasses as trap crops (pull), farmers achieved significant reductions in infestation and increased yields [74]. Integrating entomopathogenic fungi into these systems could add another layer of control, as their ability to cause mortality, inhibit feeding, and reduce reproduction would complement the deterrent and trap functions of push-pull. This diversification of tactics reduces reliance on synthetic insecticides and lowers the risk of pest adaptation, thereby enhancing the resilience of maize-based agroecosystems.

Overall, both *P. lilacinum* and *C. rosea* demonstrate strong potential as biological control agents against *S. frugiperda*, with efficacy tightly linked to spore concentration and insect developmental stage. Eggs and neonates remain the most susceptible, with LD_50_ values often an order of magnitude lower than later instars, and reliable control typically requiring ≥ 10^7^ conidia mL^-1^ within 5–7 days. These results highlight the importance of early intervention and the consideration of multiple outcomes when evaluating fungal biocontrol agents.

## Conclusion

This study demonstrates that native isolates of *P. lilacinum* and *C. rosea* possess significant potential as biological control agents against *S. frugiperda* in Tanzania. Their efficacy was strongly influenced by the interaction between spore concentration and time after treatment (DAT), indicating that the impact of spore dose depends on exposure duration, with mortality increasing over time at higher concentrations. In addition, spore concentration, insect developmental stage, and DAT each exerted significant independent effects, with eggs and neonates consistently showing higher susceptibility than later instars. Beyond direct mortality, both fungi induced sublethal effects such as reduced feeding, which could further contribute to crop protection. These findings broaden the spectrum of entomopathogenic fungi available for integrated pest management (IPM) and highlight the value of exploiting locally adapted strains to reduce reliance on chemical insecticides. Future research should focus on validating these findings under African field conditions to confirm persistence and performance under variable agroecological conditions and to integrate molecular and biochemical tools to better understand host-pathogen interactions. Moreover, evaluating compatibility with other agroecological practices such as parasitoids, intercropping, and push-pull systems will be critical for designing resilient and farmer-friendly IPM strategies. By advancing the use of indigenous fungal resources, this work provides a foundation for sustainable, environmentally safe, and context-specific management of fall armyworm in maize-based smallholder systems.

## Supporting information

S1 Table Probit analysis of *S. frugiperda* larval mortality in response to spore concentration and time after treatment of EPF*s.* Values include estimates of lethal dose (LD_50_), and median lethal time (P_50_).
(DOCX)

S1 Fig Pathogenic fungal colonies and *Spodoptera frugiperda* larvae showing signs of mycosis caused by entomopathogenic fungi: (a) *C. rosea* colony (b) *P. lilacinum* colony (c) Larva infected with *C. rosea* at 3 DAT, showing exoskeleton shedding; (d) Dead larva treated with *C. rosea* at 5 DAT; and (e) dead larva treated with *P. lilacinum* at 7 DAT. (TIF)

## References

[1] Goergen G, Kumar PL, Sankung SB, Togola A, Tamò M. First Report of Outbreaks of the Fall Armyworm Spodoptera frugiperda (J E Smith) (Lepidoptera, Noctuidae), a New Alien Invasive Pest in West and Central Africa. PLoS One. 2016;11(10):e0165632. doi: 10.1371/journal.pone.0165632 27788251 PMC5082806

[2] Food Nations AO of the U. Integrated management of the Fall Armyworm on maize: A guide for farmer field schools in Africa. Rome: FAO. 2018.

[3] Muniale FM, Mtakwa PW, Muyekho FN, Baanda AS, Massonga C. The role of conservation agriculture in management of fall army worm Spodoptera frugiperda in southern Tanzania. 2018.

[4] Kumela T, Simiyu J, Sisay B, Likhayo P, Mendesil E, Gohole L, et al. Farmers’ knowledge, perceptions, and management practices of the new invasive pest, fall armyworm (Spodoptera frugiperda) in Ethiopia and Kenya. International J Pest Management. 2018;65(1):1–9. doi: 10.1080/09670874.2017.1423129

[5] Day R, Abrahams P, Bateman M, Beale T, Clottey V, Cock M, et al. Fall Armyworm: Impacts and Implications for Africa. outlook pest man. 2017;28(5):196–201. doi: 10.1564/v28_oct_02

[6] Sisay B, Tefera T, Wakgari M, Ayalew G, Mendesil E. The Efficacy of Selected Synthetic Insecticides and Botanicals against Fall Armyworm, Spodoptera frugiperda, in Maize. Insects. 2019;10(2):45. doi: 10.3390/insects10020045 30717302 PMC6410260

[7] Kiva FM, Tryphone GM, Rwegasira GM. Response of Spodoptera frugiperda Larval Instars to Commonly Used Insecticides in Tanzania. APRJ. 2022;:1–12. doi: 10.9734/aprj/2022/v10i4195

[8] Bamisile BS, Akutse KS, Siddiqui JA, Xu Y. Model Application of Entomopathogenic Fungi as Alternatives to Chemical Pesticides: Prospects, Challenges, and Insights for Next-Generation Sustainable Agriculture. Front Plant Sci. 2021;12:741804. doi: 10.3389/fpls.2021.741804 34659310 PMC8514871

[9] Ngangambe MH, Mwatawala MW. Effects of entomopathogenic fungi (EPFs) and cropping systems on parasitoids of fall armyworm (Spodoptera frugiperda) on maize in eastern central, Tanzania. Biocontrol Science and Technology. 2020;30(5):418–30. doi: 10.1080/09583157.2020.1726878

[10] Simon E, Kudra AB, Mwatawala MW. Effects of biopesticides on developmental biology of fall armyworm (Spodoptera frugiperda (JE Smith)(Lepidoptera: Noctuidae) in maize crops in Morogoro, Tanzania. Tanzania J Agricultural Sciences. 2024;23:1–9.

[11] Butt TM, Coates CJ, Dubovskiy IM, Ratcliffe NA. Entomopathogenic Fungi: New Insights into Host-Pathogen Interactions. Adv Genet. 2016;94:307–64. doi: 10.1016/bs.adgen.2016.01.006 27131329

[12] Litwin A, Nowak M, Różalska S. Entomopathogenic fungi: unconventional applications. Rev Environ Sci Biotechnol. 2020;19:23–42. doi: 10.1007/s11157-020-09525-1

[13] Mora MAE, Castilho AMC, Fraga ME. Classification and infection mechanism of entomopathogenic fungi. Arq Inst Biol. 2018;84(0). doi: 10.1590/1808-1657000552015

[14] Paschapur A, Subbanna ARNS, Singh AK, Jeevan B, Stanley J, Rajashekhar H, et al. Unraveling the Importance of Metabolites from Entomopathogenic Fungi in Insect Pest Management. Sustainability in Plant and Crop Protection. Springer International Publishing. 2021. p. 89–120. doi: 10.1007/978-3-030-67231-7_5

[15] Shah PA, Pell JK. Entomopathogenic fungi as biological control agents. Appl Microbiol Biotechnol. 2003;61(5–6):413–23. doi: 10.1007/s00253-003-1240-8 12764556

[16] Maina UM, Galadima IB, Gambo FM, Zakaria D. A review on the use of entomopathogenic fungi in the management of insect pests of field crops. J Entomol Zool Stud. 2018;6:27–32.

[17] Zimmermann G. Review on safety of the entomopathogenic fungi Beauveria bassiana and Beauveria brongniartii. Biocontrol Sci Technol. 2007;17(6):553–96. doi: 10.1080/09583150701309006

[18] Shehzad M, Tariq M, Siddiqui JA. Entomopathogenic fungi: Natural biocontrol of insects, challenges under climate change, advancements and future prospects in Modern Agriculture. Acta Trop. 2025;269:107751. doi: 10.1016/j.actatropica.2025.107751 40691962

[19] Vivekanandhan P, Alford L, Krutmuang P. Role of entomopathogenic fungi in sustainable agriculture. Frontiers in Microbiology. 2024;:1504175. 39741596 10.3389/fmicb.2024.1504175 PMC11685110

[20] Gielen R, Ude K, Kaasik A, Põldmaa K, Teder T, Tammaru T. Entomopathogenic fungi as mortality agents in insect populations: a review. Ecology and Evolution. 2024;14:e70666. doi: 10.1002/ece3.70666 PMC11620982 39650537

[21] Akutse KS, Kimemia JW, Ekesi S, Khamis FM, Ombura OL, Subramanian S. Ovicidal effects of entomopathogenic fungal isolates on the invasive Fall armyworm Spodoptera frugiperda (Lepidoptera: Noctuidae). J Applied Entomology. 2019;143(6):626–34. doi: 10.1111/jen.12634

[22] Idrees A, Afzal A, Qadir ZA, Li J. Virulence of entomopathogenic fungi against fall armyworm, Spodoptera frugiperda (Lepidoptera: Noctuidae) under laboratory conditions. Front Physiol. 2023;14:1107434. doi: 10.3389/fphys.2023.1107434 36969609 PMC10031024

[23] Junitor A. Effects of potent fungal-based biopesticides on promising indigenous fall armyworm (Spodoptera frugiperda) (J E Smith) (Lepidoptera: Noctuidae) associated parasitoids in Kenya. 2021. https://ugspace.ug.edu.gh/server/api/core/bitstreams/1eaa4f98-dbaa-4178-8663-0b13c6705a0d/content

[24] Lugendo AR, Ben Fekih I, Caparros Megido R, Pierreux J, Francis F, Segers A. Efficacy of Entomopathogenic Fungi Against Bruchus rufimanus (Coleoptera: Chrysomelidae) in Laboratory and Field Trials Using Dropleg Spraying Technique. Agriculture. 2025;15(4):376. doi: 10.3390/agriculture15040376

[25] Masoudi A, Koprowski JL, Bhattarai UR, Wang D. Elevational distribution and morphological attributes of the entomopathogenic fungi from forests of the Qinling Mountains in China. Appl Microbiol Biotechnol. 2018;102(3):1483–99. doi: 10.1007/s00253-017-8651-4 29189901

[26] Mekonnen MA, Emirie GA, Mitiku SY, Hailemariam BN, Mekonnen MB, Mengistu AA. Psyche: A J Entomology. 2024;2024:1–8. doi: 10.1155/2024/7444094

[27] Munywoki J, Omosa LK, Subramanian S, Mfuti DK, Njeru EM, Nchiozem-Ngnitedem V-A, et al. Laboratory and Field Performance of Metarhizium anisopliae Isolate ICIPE 41 for Sustainable Control of the Invasive Fall Armyworm Spodoptera frugiperda (Lepidoptera: Noctuidae). Agronomy. 2022;12(11):2636. doi: 10.3390/agronomy12112636

[28] M Z, Fiaz M, Afzal M. Entomopathogenicity of three muscardine fungi, Beauveria bassiana, Isaria fumosorosea and Metarhizium anisopliae, against the Asian citrus psyllid, Diaphorina citri Kuwayama (Hemiptera: Psyllidae). Egyptian J Biological Pest Control. 2017;27. https://search.ebscohost.com/login.aspx?direct=true&profile=ehost&scope=site&authtype=crawler&jrnl=11101768&AN=124258445&h=Xp9k2Joasp31kvWcdHAd3p8K%2BuODt0n3L3urAEsiAMMEKsmfbeKTdMfVMgi6goAVa18RGw%2BTqu2SgRn8VGTIkA%3D%3D&crl=c

[29] Geiger A, Karácsony Z, Geml J, Váczy KZ. Mycoparasitism capability and growth inhibition activity of Clonostachys rosea isolates against fungal pathogens of grapevine trunk diseases suggest potential for biocontrol. PLoS One. 2022;17(9):e0273985. doi: 10.1371/journal.pone.0273985 36067200 PMC9447919

[30] Liu Z, Liu F-F, Li H, Zhang W-T, Wang Q, Zhang B-X, et al. Virulence of the Bio-Control Fungus Purpureocillium lilacinum Against Myzus persicae (Hemiptera: Aphididae) and Spodoptera frugiperda (Lepidoptera: Noctuidae). J Econ Entomol. 2022;115(2):462–73. doi: 10.1093/jee/toab270 35089348

[31] Ren H-Y, Liu F-F, Liang C-P, Rao X-J. Isolation of a native strain of Clonostachys rosea with insecticidal, antiphytopathogenic, and endophytic activities. Arch Microbiol. 2025;207(10):240. doi: 10.1007/s00203-025-04442-9 40875030

[32] Santos CHB, De Andrade LA, Frezarin ET, Sales LR, Rigobelo ECC. Purpureocillium lilacinum for biocontrol, bioremediation and biofertilization. https://www.preprints.org/frontend/manuscript/24b9eea7a5120377a433ac5f1fb13b65/download_pub. 2023. Accessed 2025 September 1.

[33] Toledo-Hernández RA, Toledo J, Valle-Mora J, Holguín-Meléndez F, Liedo P, Huerta-Palacios G. Pathogenicity and Virulence of Purpureocillium lilacinum (Hypocreales: Ophiocordycipitaceae) on Mexican Fruit Fly Adults. Florida Entomologist. 2019;102(2):309. doi: 10.1653/024.102.0204

[34] Funck Jensen D, Dubey M, Jensen B, Karlsson M. Clonostachys rosea to control plant diseases. https://library.oapen.org/handle/20.500.12657/61518. 2022. Accessed 2025 September 2.

[35] Nguyen HC, Tran TVA, Nguyen QL, Nguyen NN, Nguyen MK, Nguyen NTT, et al. Newly isolated Paecilomyces lilacinus and Paecilomyces javanicus as novel biocontrol agents for Plutella xylostella and Spodoptera litura. 2017. https://dro.deakin.edu.au/articles/journal_contribution/Newly_isolated_Paecilomyces_lilacinus_and_Paecilomyces_javanicus_as_novel_biocontrol_agents_for_Plutella_xylostella_and_Spodoptera_litura/20623191/1

[36] Nguyen Thi H, Nguyen QN, Dang Thi NQ, Nguyen NL, Do AD. Mass production of entomopathogenic fungi Purpureocillium lilacinum PL1 as a biopesticide for the management of Amrasca devastans (Hemiptera: Cicadellidae) in okra plantation. Egypt J Biol Pest Control. 2023;33(1). doi: 10.1186/s41938-023-00730-y

[37] Riaz M, Chen W-H, Kafle L, Tseng M-N. Morphological and molecular characterization of Purpureocillium lilacinum along with its biopesticidal effect against fall armyworm (Spodoptera frugiperda) in Southern Taiwan. Egypt J Biol Pest Control. 2024;34(1). doi: 10.1186/s41938-024-00830-3

[38] Mohammed AA, Ahmed FA, Younus AS, Kareem AA, Salman AM. Molecular identification of two entomopathogenic fungus Clonostachys rosea strains and their efficacy against two aphid species in Iraq. J Genet Eng Biotechnol. 2022;20(1):67. doi: 10.1186/s43141-022-00347-y 35482261 PMC9051009

[39] Tamta AK, Pandey R, Sharma JR, Rai R, Barman M, M G D, et al. First Record of Clonostachys rosea (Ascomycota: Hypocreales) Entomopathogenic Fungus in the Mango Hopper Amritodus atkinsoni (Hemiptera: Cicadellidae). Pathogens. 2022;11(12):1447. doi: 10.3390/pathogens11121447 36558781 PMC9781130

[40] Yang Z, Wu Q, Fan J, Huang J, Wu Z, Lin J, et al. Effects of the entomopathogenic fungus Clonostachys rosea on mortality rates and gene expression profiles in Diaphorina citri adults. J Invertebr Pathol. 2021;179:107539. doi: 10.1016/j.jip.2021.107539 33508316

[41] Nkuwi EI, RWEGASIRA G, CHILAGANE L. Occurrence of the entomopathogenic Fungi of Spodoptera frugiperda (Lepidoptera: Noctuidae) in selected areas of Tanzania. EAJSTI. 2023;4. doi: 10.37425/eajsti.v4i3.691

[42] Quandt CA, Kepler RM, Gams W, Araújo JPM, Ban S, Evans HC, et al. Phylogenetic-based nomenclatural proposals for Ophiocordycipitaceae (Hypocreales) with new combinations in Tolypocladium. IMA Fungus. 2014;5(1):121–34. doi: 10.5598/imafungus.2014.05.01.12 25083412 PMC4107890

[43] Baron NC, de Souza Pollo A, Rigobelo EC. Purpureocillium lilacinum and Metarhizium marquandii as plant growth-promoting fungi. PeerJ. 2020;8:e9005. doi: 10.7717/peerj.9005 32518715 PMC7261125

[44] Chen H, Xu J, Shao D, Zhao C, Xu X, Xu X, et al. Growth Promotion of Rice and Arabidopsis thaliana by Volatile Organic Compounds Produced by Endophytic Clonostachys Species. J Fungi (Basel). 2024;10(11):754. doi: 10.3390/jof10110754 39590673 PMC11595561

[45] Boni SB, Mwashimaha RA, Mlowe N, Sotelo-Cardona P, Nordey T. Efficacy of indigenous entomopathogenic fungi against the black aphid, Aphis fabae Scopoli under controlled conditions in Tanzania. Int J Trop Insect Sci. 2020;41(2):1643–51. doi: 10.1007/s42690-020-00365-8

[46] Burgess ND, Butynski TM, Cordeiro NJ, Doggart NH, Fjeldså J, Howell KM, et al. The biological importance of the Eastern Arc Mountains of Tanzania and Kenya. Biological Conservation. 2007;134(2):209–31. doi: 10.1016/j.biocon.2006.08.015

[47] Rovero F, Menegon M, Fjeldså J, Collett L, Doggart N, Leonard C, et al. Targeted vertebrate surveys enhance the faunal importance and improve explanatory models within the Eastern Arc Mountains of Kenya and Tanzania. Diversity and Distributions. 2014;20(12):1438–49. doi: 10.1111/ddi.12246

[48] Idrees A, Qadir ZA, Akutse KS, Afzal A, Hussain M, Islam W, et al. Effectiveness of Entomopathogenic Fungi on Immature Stages and Feeding Performance of Fall Armyworm, Spodoptera frugiperda (Lepidoptera: Noctuidae) Larvae. Insects. 2021;12(11):1044. doi: 10.3390/insects12111044 34821844 PMC8624455

[49] Humber RA. Identification of entomopathogenic fungi. Manual of techniques in invertebrate pathology. 2012. p. 151–87.

[50] Ismail SM. Insecticidal Activity of Entomopathogenic Fungi against the Immature Stages of Fall Armyworm. Progress in Chemical and Biochemical Research. 2024;7:271–82.

[51] Team RC. R: A language and environment for statistical computing. Vienna, Austria: R Foundation for Statistical Computing. 2016.

[52] Nkuwi EI, Rwegasira GM, Chilagane LA. Bio-Efficacy of Fusarium humuli and Fusarium incarnatum (Hypocreales: Nectriaceae) Against Larvae of Spodoptera frugiperda (J. E. Smith) (Lepidoptera: Noctuidae) Under Controlled Conditions. J Current Opinion Crop Sci,. 2023;4(2):56–67. doi: 10.62773/jcocs.v4i2.194

[53] Suzan AI, Dalia EL, Suzan MSB. Biologicalstudies on the efficacy of two native fungal pathogens against fall armyworm <i>Spodoptera frugiperda</i> (Lepidoptera: Noctuidae). Egypt J Plant Prot Res Inst. 2025;8(1):10–6. doi: 10.4314/ejppri.v8i1.2

[54] Demirci SNŞ, Altuntaş H. Entomopathogenic potential of Purpureocillium lilacinum against the model insect Galleria mellonella (Lepidoptera: Pyralidae). Environ Exp Biol. 2019;17:71–4.

[55] Desoky S, Abdelall M, Ahmed yasmein. Isolation, Identification, Evaluation of Purpureocillium Lilacinum Egyptian Isolate Toxicity Test in Vitro and Analysis Its Bioactive Products. Journal of the Advances in Agricultural Researches. 2022;27(4):602–17. doi: 10.21608/jalexu.2022.164739.1086

[56] Panyasiri C, Supothina S, Veeranondha S, Chanthaket R, Boonruangprapa T, Vichai V. Control Efficacy of Entomopathogenic Fungus Purpureocillium lilacinum against Chili Thrips (Scirtothrips dorsalis) on Chili Plant. Insects. 2022;13(8):684. doi: 10.3390/insects13080684 36005309 PMC9409067

[57] Sani I, Jamian S, Saad N, Abdullah S, Mohd Hata E, Jalinas J, et al. Identification and virulence of entomopathogenic fungi, Isaria javanica and Purpureocillium lilacinum isolated from the whitefly, Bemisia tabaci (Gennadius) (Hemiptera: Aleyrodidae) in Malaysia. Egypt J Biol Pest Control. 2023;33(1). doi: 10.1186/s41938-023-00657-4

[58] Sword GA. The endophytic fungal entomopathogens Beauveria bassiana and Purpureocillium lilacinum enhance the growth of cultivated cotton (Gossypium hirsutum) and negatively affect survival of the cotton bollworm (Helicoverpa zea). Biol Control. doi: 10.1016/j.biocontrol.2015.1016

[59] Khan M, Tanaka K. Purpureocillium lilacinum for plant growth promotion and biocontrol against root-knot nematodes infecting eggplant. PLoS One. 2023;18(3):e0283550. doi: 10.1371/journal.pone.0283550 36961807 PMC10038259

[60] Silva SD, Carneiro RMDG, Faria M, Souza DA, Monnerat RG, Lopes RB. Evaluation of Pochonia chlamydosporia and Purpureocillium lilacinum for Suppression of Meloidogyne enterolobii on Tomato and Banana. J Nematol. 2017;49(1):77–85. doi: 10.21307/jofnem-2017-047 28512379 PMC5411256

[61] Speckbacher V, Zeilinger S. Secondary metabolites of mycoparasitic fungi. Secondary metabolites-sources and applications. 2018. p. 37–55.

[62] Mohamed MAHMOUDF, Bendebbah R, Benssaci B, Toudji F, Tafifet L, Krimi Z. Entomopathogenic efficacy of the endophytic fungi: Clonostachys sp. and Beauveria bassiana on Tuta absoluta (Meyrick) (Lepidoptera: Gelechiidae) larvae under laboratory and greenhouse conditions. Egypt J Biol Pest Control. 2021;31:43. doi: 10.1186/s41938-021-00392-8

[63] Mohammed AA, Younus AS, Ali AN. Efficacy of Clonostachys rosea, as a promising entomopathogenic fungus, against coleopteran stored product insect pests under laboratory conditions. Egypt J Biol Pest Control. 2021;31(1). doi: 10.1186/s41938-021-00405-6

[64] Al-Nabhani SS, Kazerooni EA, Al-Raqmi S, Al-Hashmi M, Hussain S, Velazhahan R, et al. Isolation of Clonostachys rosea and Characterizing Its Entomopathogenic Activity against Dubas Bug (Ommatissus lybicus) Nymphs and Adults. Agriculture. 2024;14(10):1770. doi: 10.3390/agriculture14101770

[65] Anwar W, Ali S, Nawaz K, Iftikhar S, Javed MA, Hashem A, et al. Entomopathogenic fungus Clonostachys rosea as a biocontrol agent against whitefly (Bemisia tabaci). Biocontrol Science and Technology. 2018;28(8):750–60. doi: 10.1080/09583157.2018.1487030

[66] Li M, Li J, An Z, Wang S, Lai Y. First Record of Clonostachys rosea as an Entomopathogenic Fungus of the Cephus fumipennis (Hymenoptera: Cephidae) in China. Biology (Basel). 2025;14(9):1240. doi: 10.3390/biology14091240 41007384 PMC12467502

[67] Mahmoudi H, Amini A, Mirzaee MR, Sadeghi H, Tavakkoli GR. Clonostachys rosea, a new and promising entomopathogenic fungus infecting pupa of jujube fruit fly, Carpomya vesuviana. Mycologia Iranica. 2018;5:43–9.

[68] Yuan C, Wang X, Asemoloye MD, Wang Y, Gargano ML, Xue H-J, et al. First record of *Clonostachys rosea* as entomopathogenic fungus of Coleoptera in China. Plant Biosystems - An International J Dealing with all Aspects of Plant Biology. 2021;155: 1240–6. doi: 10.1080/11263504.2021.2013339

[69] Sun Z-B, Li S-D, Ren Q, Xu J-L, Lu X, Sun M-H. Biology and applications of Clonostachys rosea. J Appl Microbiol. 2020;129(3):486–95. doi: 10.1111/jam.14625 32115828

[70] Goettel MS, Glare T. 11 entomopathogenic fungi and their role in regulation of insect populations. Wallingford, UK: CAB International. 2010.

[71] Al-Ayat AA, Gharib AM, Hassuba MM, Atta AA, Mesbah HA, Gad HA. Effect of entomopathogenic nematode and fungi on mortality and development of Spodoptera frugiperda (JE Smith) larvae. Journal of Agricultural Science and Technology. 2025;27:421–33.

[72] Vega FE, Goettel MS, Blackwell M, Chandler D, Jackson MA, Keller S, et al. Fungal entomopathogens: new insights on their ecology. Fungal Ecology. 2009;2(4):149–59. doi: 10.1016/j.funeco.2009.05.001

[73] Haider MU, Ahmad S. Virulence of different entomopathogenic fungal strains against different life stages of fall armyworm, Spodoptera frugiperda (Lepidoptera: Noctuidae). Journal of Wildlife and Biodiversity. 2025;9:183–99.

[74] Midega CAO, Pittchar JO, Pickett JA, Hailu GW, Khan ZR. A climate-adapted push-pull system effectively controls fall armyworm, Spodoptera frugiperda (J E Smith), in maize in East Africa. Crop Protection. 2018;105:10–5. doi: 10.1016/j.cropro.2017.11.003

