# Supplementary material for: Pathogenicity of Purpureocillium lilacinum and Clonostachys rosea against fall armyworm (Spodoptera frugiperda) under laboratory conditions
Source: PLoS One. 2026 Mar 16;21(3):e0334730. doi: 10.1371/journal.pone.0334730 (PMC12991274; doi:10.1371/journal.pone.0334730)
Supplement: S1 Table — Values include estimates of lethal dose (LD50), and median lethal time (P50). (DOCX) [file pone.0334730.s001.docx]

**S1Table:** Probit analysis of S. frugiperda larval mortality in response to spore concentration and exposure time of entomopathogenic fungi. Values include estimates of lethal dose (LD_50_), and median lethal time (P_50_).

| Analysis | Parameter | Estimate |
| --- | --- | --- |
| Dose-based mortality | LD₅₀ (median lethal dose) | ≈1.6×10¹² conidia mL^-1^ |
|  | LD₈₅ (85% lethal dose) | ≈3.9×10¹⁶ conidia mL^-1^ |
| Time-based mortality (all doses) | LT₅₀ (median lethal time) | 15.9 DAT |
|  | LT₈₅ (85% lethal time) | 25.0 DAT |
| Time-based mortality, 10⁹ conidia mL^-1^ | LT₅₀ / LT₈₅ | 15.4 / 23.0 DAT |
|  | LT₅₀ / LT₈₅ | 13.2 / 20.4 DAT |
|  | LT₅₀ / LT₈₅ | 11.8 / 19.2 DAT |

Notes: LD = lethal dose; LT = lethal time. Estimates derived from probit dose-response and time-mortality models. DAT = days after treatment.
