# Supplementary figures and images for: Pathogenicity of Purpureocillium lilacinum and Clonostachys rosea against fall armyworm (Spodoptera frugiperda) under laboratory conditions
Source: PLoS One. 2026 Mar 16;21(3):e0334730. doi: 10.1371/journal.pone.0334730 (PMC12991274; doi:10.1371/journal.pone.0334730)

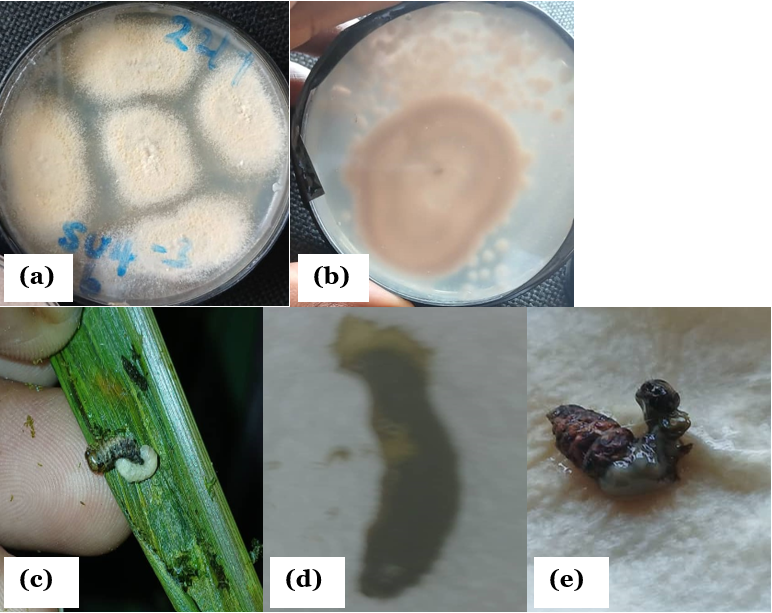

Supplement: S1 Fig — (TIF) [file pone.0334730.s002.tif]
