# Supplementary material for: Pathogenicity of Purpureocillium lilacinum and Clonostachys rosea against fall armyworm (Spodoptera frugiperda) under laboratory conditions
Source: PLoS One. 2026 Mar 16;21(3):e0334730. doi: 10.1371/journal.pone.0334730 (PMC12991274; doi:10.1371/journal.pone.0334730)
Supplement: Attachment — Submitted filename: Comments.pdf [file pone.0334730.s003.pdf]

Reviewer Comments (PONE-D-25-53276)

Decision: Accept with Minor Revisions

Specific Comments for Authors
Please address the following points to enhance the clarity and completeness of the manuscript. No additional experiments are required.

1. Abstract and Conclusion Clarity

i. **Repetitive Content:** The final paragraph of the Abstract, lines 37–43, largely repeats the detailed findings summarized in the preceding *Results* paragraph, lines 30–37. To improve conciseness, consider synthesizing the final paragraph to focus purely on the validation of the hypothesis and the significance of the findings, rather than re-stating the quantitative results.

ii. **Conclusion Sentence Refinement:** In the Conclusions section (lines 408-424), the sentence "Their efficacy was strongly influenced by spore concentration and insect developmental stage, with eggs and neonates consistently showing higher susceptibility than later instars" is highly accurate but could be enriched by briefly mentioning the crucial third factor confirmed by your analysis. Table 1 shows that **Time After Treatment (DAT)** was also a highly significant factor in larval mortality ($p < 0.001$), with a significant concentration x time interaction ($p = 0.010$). Please ensure the conclusion reflects all three major factors demonstrated in the data (Dose, Stage, and Time) for complete accuracy.

2. Methods Documentation

i. **Fungal Isolate Characterization:** The Methods section states that the EPF species were obtained from soil samples collected in the Uluguru Mountains, isolated through selective media, and screened for pathogenicity. For full reproducibility, please briefly clarify how the specific identity of the native isolates (i.e., confirming they were truly *Purpureocillium lilacinum* and *Clonostachys rosea*) was achieved. Was this identification based solely on morphology, or was a confirmatory method (such as molecular sequencing/PCR) used? This clarification should be added around lines 178-187.

ii. **Rearing Conditions for *S. frugiperda*:** The rearing conditions are well-described (25±2°C, 65±5% relative humidity, 12:12 h L:D) relative humidity, 12:12 h L:D). Please confirm in the Materials and Methods (lines 198-199) that **all bioassay test units** (larvae and eggs treated with EPFs or control) were maintained under these identical controlled conditions throughout the 9-day observation period, as consistency in environmental parameters is critical for EPF bioassays.

3. Results and Discussion Context

i. **LD50 Value Context:** The Results section reports that the overall lethal dose (LD50) was estimated at 1.6×10^{12} conidia mL^{-1}. While the manuscript correctly notes that *second and third instars* required lower concentrations to reach LD50 (Fig 3), the single combined LD50 value of 1.6×10^{12} is significantly higher than most reported values for EPFs against early-stage lepidopterans (e.g., 10^6 - 10^8 conidia mL^{-1} cited in the Discussion for similar

assays). Please confirm in the text (around lines 268–269) that this high calculated LD50 (1.6×10^{12}) represents the average estimated virulence across all tested instars (L2 to L5), which includes the significantly less susceptible late instars, thus explaining the high magnitude. This clarification will prevent potential misinterpretation of the strain's overall efficacy.

ii. **Justification for Instar Selection:** The rationale for excluding the 1st and 6th instars is provided in lines 202-207. Please move this detailed justification to the end of the *Collection and rearing of test insect* subsection (around line 176), or within the *Bioassay* subsection, rather than placing it after the description of the mortality measurement protocols (lines 199-202). This slight reorganization will improve the flow of the Methods section.

4. Figure and Table Presentation

i. **Figure 1 Legend Clarity:** Figure 1 shows the effect of concentration and time on mortality. Table 1 shows that *both* Concentration and DAT were highly significant. The legend for Fig 1 states: "Different letters above bars indicate significant differences among concentrations (Tukey's HSD, $p < 0.05$)". Please ensure the figure itself (or its associated legend/notes) also clearly communicates the significance found across the **Days After Treatment (DAT)**, as this is a fundamental and highly significant finding ($p < 0.001$).

Overall Assessment

This is a valuable contribution to the literature on biological control in Sub-Saharan Africa. The minor revisions requested aim only to strengthen the presentation and ensure that the powerful statistical conclusions are fully supported by clear text and methodology.
