# Supplementary material for: Pathogenicity of Purpureocillium lilacinum and Clonostachys rosea against fall armyworm (Spodoptera frugiperda) under laboratory conditions
Source: PLoS One. 2026 Mar 16;21(3):e0334730. doi: 10.1371/journal.pone.0334730 (PMC12991274; doi:10.1371/journal.pone.0334730)
Supplement: Attachment — Submitted filename: Response to Reviewers.docx [file pone.0334730.s004.docx]

**Rebuttal letter**

**Reviewer comment 1**

“Please ensure that your manuscript meets PLOS ONE's style requirements, including those for file naming. The PLOS ONE style templates for formatting sample main body and formatting sample title author affiliations.pdf”.

**Response 1:** We thank the reviewer and the Editorial Office for this comment. We have carefully revised the manuscript to fully comply with PLOS ONE formatting and style requirements, using the official PLOS ONE templates for the main text and the title/authors/affiliations pages. The manuscript files have been correctly renamed according to PLOS ONE file-naming guidelines, and formatting has been standardized throughout (including Title and section headings, references, tables, and figure captions). We confirm that the revised submission now conforms to all PLOS ONE style requirements.

**Reviewer comment 2**
“In your Methods section, please provide additional information regarding the permits you obtained for the work. Please ensure you have included the full name of the authority that approved the field site access and, if no permits were required, a brief statement explaining why.”

**Response 2:** We have revised the Methods section (Ethics statement) to clearly state the full name of the approving authority and to clarify field site access (lines 232-241). Specifically, we now indicate that the study protocol was approved by the Ethics Review Board of the College of Agriculture, Sokoine University of Agriculture (SUA), Tanzania, and that permission to access and conduct research at the field sites and laboratory was granted by Sokoine University of Agriculture, which owns and manages all land and facilities where the study was conducted. We also clarify that no additional permits from external authorities were required because the research was carried out entirely on university-owned property.

**Reviewer comment 3**

It appears that at least parts of the introduction is AI generated according to copyleaks AI detector. There should be a declaration at the end of the MS in the Acknowledgements as outlined by the journal

**Response 3:** We confirm that AI tools (ChatGPT, OpenAI) were used only for grammar checking and reducing redundancy in the Introduction. All text was reviewed, revised, and approved by the authors. In accordance with PLOS ONE guidelines, we have added a declaration in the Acknowledgements to clarify this use (lines 461-464).

**Reviewer comment 4**

line 195: please describe how was the treatment carried out and how long were the larvae exposed to the spores of the different fungi, and when were the paper towels exchanged under the larvae.
Expression of the treatment in l/ha would be also useful for practicing pest managers, so they could compare the dosage to already existing products.

**Response 4:** We have revised the Materials and Methods section to explicitly confirm that all bioassay test units (larvae and eggs, including EPF-treated and control groups) were maintained under identical controlled conditions (25 ± 2 °C, 65 ± 5% RH, 12:12 h L:D) throughout the observation period. In addition, we clarified the treatment application method which was spraying using handheld sprayer, duration of larval exposure to fungal spores-sprayed only once and monitored. Paper towels were used only to absorb excess spray solution which were removed after 24 hours post treatment.

Expression of the treatment dose in L ha⁻¹ could not be meaningfully provided, as the experiment was conducted entirely under controlled laboratory conditions using a fixed spray volume per container rather than field-based application rates. We have clarified this limitation in the Methods section. Field-equivalent application rates would require validation under greenhouse or field conditions using calibrated spray equipment.

**Reviewer comment 5**

line 267: I think that the supplementary table could be included after this sentence or paragraph, because it contains relevant and valuable information.

**Response 5:** We agree with the reviewer that the supplementary table contains relevant and valuable information. In accordance with PLOS ONE guidelines, this information has been included as Supporting Information (S1 Table) and is clearly cited in the Results section immediately after the relevant sentence (Line 273), allowing readers to readily access the detailed probit model outputs.

**Reviewer comment 6**

lines 279, 289: "species related interactions" and "Neither species identity" would be more cleared if the authors would include the fungi or fungal words.
I would appreciate images of the fungal colonies the healthy and infected larvae and images of the eaten corn leaves from each treatment to make the MS more visually appealing.

**Response 6:** To improve clarity, we have revised the manuscript by explicitly referring to *fungal species* when describing species-related interactions and species identity effects. This clarification has been incorporated in the sections on larval feeding consumption and egg mortality.

In addition, representative images of fungal colonies, and healthy and infected larvae added as supplementary figures (S1Figure) to enhance the visual presentation of the study.

**Reviewer comment 7**

I. Repetitive Content: The final paragraph of the Abstract, lines 37-43, largely repeats the detailed findings summarized in the preceding Results paragraph, lines 30-37. To improve conciseness, consider synthesizing the final paragraph to focus purely on the validation of the hypothesis and the significance of the findings, rather than re-stating the quantitative results.
ii. Conclusion Sentence Refinement: In the Conclusions section (lines 408-424), the sentence "Their efficacy was strongly influenced by spore concentration and insect developmental stage, with eggs and neonates consistently showing higher susceptibility than later instars" is highly accurate but could be enriched by briefly mentioning the crucial third factor confirmed by your analysis. Table 1 shows that Time After Treatment (DAT) was also a highly significant factor in larval mortality (p < 0.001), with a significant concentration x time interaction (p = 0.010). Please ensure the conclusion reflects all three major factors demonstrated in the data (Dose, Stage, and Time) for complete accuracy.

**Response 7:** The final paragraph of the Abstract has been revised to remove repetition of quantitative results and instead synthesize the findings in terms of hypothesis validation and broader significance. The revised text now emphasizes the primary drivers of fungal efficacy and the implications for integrated pest management.

The Conclusions section has been revised to explicitly incorporate time after treatment (DAT) as a third major factor influencing fungal efficacy, alongside spore concentration and insect developmental stage. The revised text now accurately reflects the significant effects of exposure time and the concentration × time interaction demonstrated in the analysis, ensuring full consistency between the Results and Conclusions.

**Reviewer comment 8**

Fungal Isolate Characterization: The Methods section states that the EPF species were obtained from soil samples collected in the Uluguru Mountains, isolated through selective media, and screened for pathogenicity. For full reproducibility, please briefly clarify how the specific identity of the native isolates (i.e., confirming they were truly Purpureocillium lilacinum and Clonostachys rosea) was achieved. Was this identification based solely on morphology, or was a confirmatory method (such as molecular sequencing/PCR) used? This clarification should be added around lines 178-187.

**Response 8:** We have clarified the identification procedure of the native entomopathogenic fungal isolates by stating that species identity was confirmed using both morphological characteristics and molecular analysis based on ITS rDNA sequencing, with sequences compared against GenBank references (Lines 183-187).

**Reviewer comment 9**

Results and Discussion Context
i. LD50 Value Context: The Results section reports that the overall lethal dose (LD50) was estimated at 1.6 x 1012 conidia mL-1. While the manuscript correctly notes that second and third instars required lower concentrations to reach LD50 (Fig 3), the single combined LD50 value of 1.6 x 1012 is significantly higher than most reported values for EPFs against early-stage lepidopterans (e.g., 106 - 108 conidia mL-1 cited in the Discussion for similar assays). Please confirm in the text (around lines 268–269) that this high calculated LD50 (1.6 x 1012) represents the average estimated virulence across all tested instars (L2 to L5), which includes the significantly less susceptible late instars, thus explaining the high magnitude. This clarification will prevent potential misinterpretation of the strain’s overall efficacy.

ii. Justification for Instar Selection: The rationale for excluding the 1st and 6th instars is provided in lines 202-207. Please move this detailed justification to the end of the Collection and rearing of test insect subsection (around line 176), or within the Bioassay subsection, rather than placing it after the description of the mortality measurement protocols (lines 199-202). This slight reorganization will improve the flow of the Methods section.

**Response 9:** We have justified that the reported overall LD50 of 1.6 × 10¹² conidia mL⁻¹ represents the average estimated virulence across all tested instars, including the less susceptible late instars. This explanation contextualizes the high magnitude and prevents misinterpretation of the strain’s efficacy (Lines 274-276).

The instar selection justification has been moved from 202-207 to around line 176-180

**Reviewer comment 10**

Figure 1 shows the effect of concentration and time on mortality. Table 1 shows that both Concentration and DAT were highly significant. The legend for Fig 1 states: "Different letters above bars indicate significant differences among concentrations (Tukey’s HSD, p < 0.05)". Please ensure the figure itself (or its associated legend/notes) also clearly communicates the significance found across the Days After Treatment (DAT), as this is a fundamental and highly significant finding (p < 0.001).

**Response 10:** We have revised the Figure 1 legend and Table 1 notes and titles to explicitly report the significance of Days After Treatment (DAT).
